# Synaptic vesicles isolated from the electric organ of *Torpedo californica* and from the central nervous system of *Mus musculus* contain small ribonucleic acids (sRNAs)

**DOI:** 10.1016/j.gdata.2017.02.015

**Published:** 2017-03-08

**Authors:** Huinan Li, Cheng Wu, Rodolfo Aramayo, Matthew S. Sachs, Mark L. Harlow

**Affiliations:** Department of Biology, Texas A&M University, TAMU 3258, College Station, TX 77843-3474, United States

**Keywords:** Synaptic vesicle, Micro-RNA, tRNA fragment, *Torpedo californica*, *Mus musculus*

## Abstract

Synaptic vesicles (SVs) are presynaptic organelles that load and release small molecule neurotransmitters at chemical synapses. In addition to classic neurotransmitters, we have demonstrated that SVs isolated from the Peripheral Nervous Systems (PNS) of the electric organ of *Torpedo californica,* a model cholinergic synapse, and SVs isolated from the Central Nervous System (CNS) of *Mus musculus* (mouse) contain small ribonucleic acids (sRNAs; ≤ 50 nucleotides) (Scientific Reports, 5:1–14(14918) Li et al. (2015) [1]). Our previous publication provided the five most abundant sequences associated with the *T. californica* SVs, and the ten most abundant sequences associated with the mouse SVs, representing 59% and 39% of the total sRNA reads sequenced, respectively). We provide here a full repository of the SV sRNAs sequenced from *T. californica* and the mouse deposited in the NCBI as biosamples. Three data studies are included: SVs isolated from the electric organ of *T. californica* using standard techniques, SVs isolated from the electric organ of *T. californica* using standard techniques with an additional affinity purification step, and finally, SVs isolated from the CNS of mouse. The three biosamples are available at https://www.ncbi.nlm.nih.gov/biosample/ SRS1523467, SRS1523466, and SRS1523472 respectively.

**Table:** 

Specifications	
Organism/cell line/tissue	*Tetronarce californica* (formally *Torpedo californica*)/electric organ
Sex	female
Sequencer or array type	Illumina HiSeq 2500
Data format	Raw data BioProject 323486 SRA Study SRS1523467
Experimental factors	Small RNA (sRNAs; ≤ 50 nucleotides) populations associated with synaptic vesicles isolated from the electric organ.
Experimental features	Synaptic vesicles from the electric organ were isolated using standard procedures. Prior to RNA extraction, exogenous RNA (non-luminal) was removed by RNAse cocktail.
Consent	n/a
Sample source location	San Pedro, California, USA

Organism/cell line/tissue	*Tetronarce californica* (formally *Torpedo californica*)/electric organ
Sex	female
Sequencer or array type	Illumina HiSeq 2500
Data format	Raw data BioProject 323486 SRA Study SRS1523466
Experimental factors	Small RNA (sRNAs; ≤ 50 nucleotides) populations associated with synaptic vesicles isolated from the electric organ.
Experimental features	Synaptic vesicles from the electric organ were 1) isolated using standard procedures, and 2) further purified using an immuno-affinity procedure. Prior to RNA extraction, exogenous RNA (non-luminal) was removed by RNAse cocktail.
Consent	n/a
Sample source location	San Pedro, California, USA

Organism/cell line/tissue	*Mus musculus*/brain
Sex	Pooled male and female
Sequencer or array type	Illumina HiSeq 2500
Data format	Raw data BioProject 323487 SRA Study SRS1523472
Experimental factors	Small RNA (sRNAs; ≤ 50 nucleotides) populations associated with synaptic vesicles isolated from the mouse brain.
Experimental features	Synaptic vesicles from the brain were isolated using standard procedures. Prior to RNA extraction, exogenous RNA (non-luminal) was removed by RNAse cocktail.
Consent	n/a
Sample source location	Winchester, Virginia, USA

## 1 Direct link to deposited data

The deposited data can be found at: https://www.ncbi.nlm.nih.gov/.

The data consists of three studies. Two studies contain the SV sRNAs isolated from the electric organ of *T. californica*. The first study contains the SV sRNAs isolated using the standard procedure (SRS1523467). The second study contains the SV sRNAs from SVs that had an additional immuno-affinity purification step prior to RNA extraction (SRS1523466). The third study contains the SV sRNAs isolated from the CNS of *M. musculus* (SRS1523472). The standard SV isolation procedure was used for the *M. musculus* tissue.

## 2 Experimental design, materials and methods

### 2.1 Isolation of synaptic vesicles

Methods were adapted from Ohsawa [2]. A Spex Freezer Mill 6800 (Spex Sample Prep; Metuchen, NJ) was cooled to − 180 ^°^C and ~ 25 g of frozen electric organ from an individual *Torpedo californica* (adult female; Aquatic Research Consultants; San Pedro, CA) or 25 g frozen *Mus musculus* brains (~ 50 Swiss Webster mouse brains of both male & female; BioChemed Services, Winchester, VA) was ground with 25 g of frozen buffer pellets (320 mM Sucrose, 10 mM TRIS-Cl, pH 7.4) Sigma-Aldrich; St. Louis, MO). The resulting powder of buffer and electric organ/brain was warmed to 4 ^°^C with 50 ml of buffer solution (320 mM Sucrose, 10 mM Tris-Cl, pH 7.4, 4 ^°^C). The resulting slurry (100 ml) was centrifuged at 20,000 rpm for 10 min (Beckman Coulter JA-20 rotor - Avanti J25 centrifuge) (Beckman Coulter; Brea, CA). The resulting supernatant was centrifuged at 34,000 rpm for 40 min (70ti rotor - Optima X80; Beckman). The supernatant was then loaded onto a 4 ml/4 ml 0.6 M/1.2 M sucrose step gradient (10 mM Tris-Cl, pH 7.4), then centrifuged at 48,000 rpm for 2 h (70ti rotor - Optima X80). The 4 ml 0.6 M (1.07 g/ml density) sucrose fluffy layer, enriched in vesicles, was collected. Heavier densities and pellet (> 0.6 M sucrose), known to be enriched in exosomes, were discarded [3], [4]. A 2 ml sample of enriched vesicles was filtered using a 0.22 μm spin column (Spin-x, Corning; Corning, NY) to remove any large debris. The filtrate was injected into a Pharmacia LC500 plus FPLC (GE Healthcare, Fairfield, CT) and run through a 25 cm 4% agarose bead column (Bioscience Beads; West Warwick, RI). Separate bead columns were prepared for electric organ and mouse brains to ensure no contamination. The FPLC was eluted with a buffer solution (0.2 M NaCl, 10 mM HEPES, pH 7.4; Sigma) at a flow rate of 1.0 ml/min. The second major peak was collected, and the vesicles concentrated to a protein concentration of 5 mg/ml for RNA isolation (measured by Bradford Assay) (Bio-Rad Laboratories, Inc.; Hercules, CA) using a Stirred Cell apparatus with a 100 kDa filter (PLHK02510; EMD Millipore, Billerica, MA). As a further enrichment, SVs from one preparation of *T. californica* were affinity enriched using dynabeads (100.07D; Dynabeads; Invitrogen/Life Technologies, Carlsbad, CA) with VAChT antibody (ab68986; Abcam; Cambridge, England) [5], [6].

### 2.2 Isolation of synaptic vesicle sRNAs and sequencing

SVs were isolated and concentrated as described above. Each sample consisted of 50 μl of SVs (5 mg/ml) in PBS (80 mM Na_2_HPO_4_ and 25 mM NaH_2_PO_4_, 100 mM NaCl pH 7.4; Sigma). To remove exogenous RNA, each SV preparation was treated with 50 μl PBS pH 7.4 with 1 μl RNase cocktail as instructed (RNase A (500 U ⁄ ml) and RNase T1 (20,000 U ⁄ ml), AM2286; Ambion/Life Technologies). SV RNA was extracted after the treatments using 900 μl TRIzol (Invitrogen/Life Technologies) followed by 200 μl chloroform and 400 μl isopropanol (EMD Millipore), with a final precipitation by 75% ethanol. Library preparation and sequencing were performed by the Genomic Sequencing and Analysis Facility at the University of Texas, Austin. An Agilent 2100 (Agilent Technologies, Santa Clara, CA) was used for quality control. The sRNAs were prepared for sequencing using the TruSeq small RNA sample preparation kit (Illumina; San Diego, CA). Single-end reads (100 bp) were sequenced on an Illumina HiSeq 2500(Illumina).

## 3 Discussion

Disruptions in the normal maintenance of chemical synapses, or in the processes by which chemical synapses are reorganized during memory formation, are implicated in a wide range of neurological diseases. A vital aspect of normal synaptic function is the proper post-transcriptional regulation of protein synthesis at sites away from the nerve cell body. This local protein synthesis at the synapse is regulated by activity, and requires a host of mRNAs, translation factors, and ribosomes [7], [8], [9], [10], [11]. In addition, it is suspected that microRNA (miRNA) and other non-coding RNA (ncRNA) that include, but are not restricted to, endogenous small interfering RNA (esiRNAs), piwi-interacting RNA (piRNA), antisense and long-ncRNA, play a key role in regulating translation [12]. Activity at chemical synapses is controlled by the fusion of SVs with the presynaptic plasma membrane, and the release of the vesicles' contents. We hypothesize that, in addition to neurotransmitters, SVs contain small RNAs (sRNAs; ≤ 40 nucleotides), and in a previous paper provided evidence for the presynaptic origin and luminal presence for the most abundant SV sRNAs [1]. The SV sRNA data presented in this work represent the entirety of the data sets collected for the previous study, including microRNAs associated with the SVs isolated from the electric organ of *T. californica* not discussed in the previous publication [1]. The data sets produced in these studies have been deposited in the NCBI: SRS1523467, SRS1523466, SRS1523472.

## Conflict of interest

The authors declare no competing financial interests.
